# Lipoid Pneumonia From Bug Spray: A Case Report

**DOI:** 10.7759/cureus.109444

**Published:** 2026-05-22

**Authors:** David Wang, Kurian Chandy, Sarah Steenson, Hanna Nale, Wedad Alfarkh

**Affiliations:** 1 Internal Medicine, University of Missouri School of Medicine, Columbia, USA; 2 Pulmonary and Critical Care, University of Missouri School of Medicine, Columbia, USA; 3 Pathology and Laboratory Medicine, University of Missouri School of Medicine, Columbia, USA; 4 Pathology, University of Missouri School of Medicine, Columbia, USA

**Keywords:** bal lavage, bronchoscopy, exogenous lipoid pneumonia, ground-glass opacities, systemic steroids

## Abstract

Exogenous lipoid pneumonia is an uncommon inflammatory lung disease caused by the aspiration or inhalation of lipid-containing substances. Clinical presentation is highly variable, ranging from asymptomatic disease to progressive respiratory failure, and the condition is often underrecognized in clinical practice. Diagnosis can be challenging because of nonspecific symptoms and imaging findings. We report a case of a 38-year-old woman with suspected exogenous lipoid pneumonia associated with exposure to a commercially available bug spray containing plant-based oils. Bronchoscopy with bronchoalveolar lavage revealed eosinophilia and a markedly elevated lipid-laden macrophage index, supporting the diagnosis of lipoid pneumonia. The patient was treated with systemic corticosteroids and advised to avoid further exposure, resulting in clinical improvement and near-complete radiographic resolution on follow-up imaging. This case highlights the importance of obtaining a detailed exposure history in patients with persistent respiratory symptoms, as recognition of lipid exposure can facilitate timely diagnosis and targeted management.

## Introduction

Lipoid pneumonia is an uncommon inflammatory lung disease that develops when fats and oils accumulate within the alveoli, the alveolar air spaces [1]. When these lipids originate from outside the body, such as from aspirated or inhaled oil-based substances, the condition is classified as exogenous lipoid pneumonia [2]. Although rare, exogenous lipoid pneumonia has been associated with a wide range of exposures and may be underrecognized in clinical practice because of its nonspecific presentation [3]. Reported exposures include electronic cigarettes, petroleum-based ointments, lubricants used by automobile mechanics, furniture polish, and cutting oils [4].

The pathophysiology involves deposition of lipid material within the distal airways. Alveolar macrophages engulf the lipid material but are unable to effectively metabolize it. This results in repeated release of lipids following macrophage death, persistent inflammation, and, in some cases, a granulomatous reaction that can impair gas exchange [5].

The clinical presentation is highly variable. While some individuals remain asymptomatic, others may develop significant respiratory illness [6]. When symptoms occur, cough and shortness of breath are the most common complaints, although patients may also report chest discomfort, intermittent fever, hemoptysis, or unintentional weight loss [7]. Radiologic findings are often nonspecific and may mimic other pulmonary conditions, including infectious pneumonia or malignancy, further complicating diagnosis [4].

On physical examination, lung auscultation may reveal crackles or wheezing; however, many affected individuals have normal examination findings [8]. There are no universally established diagnostic criteria for lipoid pneumonia. Diagnosis typically relies on a combination of a compatible exposure history, supportive radiologic findings, and confirmation of lipid-laden macrophages or fatty deposits on tissue biopsy or bronchoalveolar lavage (BAL) [9]. Prognosis depends on the underlying etiology, patient age, timeliness of diagnosis, and associated medical comorbidities. Treatment focuses on eliminating the inciting exposure, providing supportive care, administering corticosteroids, and considering lavage in patients refractory to standard therapy [4]. We present the case of a patient evaluated at the University of Missouri Hospital who likely developed lipoid pneumonia following exposure to a specific type of bug spray.

This article was previously presented as a poster at the 2025 CHEST Conference on October 21, 2025.

## Case presentation

The patient was a 38-year-old woman with a medical history of multiple sclerosis, migraines, pulmonary embolism, hypertension, major depressive disorder, and methylenetetrahydrofolate reductase mutation who presented to the hospital with dyspnea, chest pain, and malaise. Her home medications included acetaminophen-hydrocodone, albuterol, atenolol, fluoxetine, gabapentin (for multiple sclerosis), hydroxyzine, oxybutynin, prazosin, promethazine, tamsulosin, trazodone, and verapamil.

Her symptoms began approximately two months before admission, around the time she moved into a new apartment. During that period, her furniture had been sprayed with BugMD, although the exact duration of her exposure in the apartment was unknown. She denied cigarette smoking and recent travel history but reported marijuana use. Over the two months preceding admission, she presented to the emergency department multiple times with symptoms including headache, dyspnea, sore throat, and cough. Workup during those visits was notable for an elevated absolute eosinophil count (AEC) of up to 4,130 cells/µL. A chest CT pulmonary angiography protocol performed during those earlier visits showed no acute findings. She was treated with inhalers, corticosteroids, and antibiotics, with minimal improvement in symptoms.

Physical examination on admission was notable for diffuse bilateral wheezing and tachycardia but was otherwise unremarkable. Laboratory studies revealed a white blood cell (WBC) count of 17.05 K/µL, AEC of 1,140 cells/µL, hemoglobin of 11.9 g/dL, platelet count of 426 K/µL, sodium of 138 mmol/L, potassium of 3.8 mmol/L, glucose of 120 mg/dL, creatinine of 0.6 mg/dL, and normal liver function tests (Table 1). Troponin level was within normal limits. Urinalysis showed moderate leukocyte esterase, 0-3 WBCs/hpf, 3-6 RBCs/hpf, and numerous epithelial cells but was otherwise unremarkable. Venous blood gas analysis demonstrated a normal pH without evidence of CO₂ retention. The Fungitell test was negative, and HIV testing was nonreactive. An autoimmune workup, including fluorescent antinuclear antibody (FANA), antineutrophil cytoplasmic antibodies (ANCA), rheumatoid factor, anti-myeloperoxidase (MPO), and anti-proteinase 3 (PR3), was unremarkable. C-reactive protein (CRP) was elevated at 3.4 mg/dL. Chest radiography demonstrated no acute cardiopulmonary abnormalities (Figure 1). Computed tomography pulmonary angiography showed no evidence of acute pulmonary embolism but revealed multifocal bilateral ground-glass opacities with upper lobe predominance (Figure 2). She received one dose of IV methylprednisolone (Solu-Medrol) 50 mg and was started on levofloxacin for empiric treatment of pneumonia. Blood cultures obtained on the day of admission showed no growth.

**Table 1:** Notable labs upon admission including reference ranges UA: urinalysis, WBC: white blood cell, RBC: red blood cells, hpf: high-power field, pCO₂: partial pressure of carbon dioxide, CRP: C-reactive protein, HIV: human immunodeficiency virus, FANA: fluorescent antinuclear antibody, ANCA: antineutrophil cytoplasmic antibodies, RF: rheumatoid factor, IgM: immunoglobulin M, MPO: myeloperoxidase, PR3: proteinase 3, NA: not applicable, AEC: absolute eosinophil count

Lab	Lab value	Reference range
Sodium	138	136-145 nmol/L
Potassium	3.8	3.5-5.1 mmol/L
Aspartate aminotransferase	14	<34 units/L
Alanine aminotransferase	24	10-50 units/L
Total bilirubin	0.6	0.3-1.2 mg/dL
Creatinine	0.6	0.7-1.2 mg/dL
Glucose	120	70-139 mg/dL
WBC	17.05	3.5-10.5K/uL
AEC	1140	50-500/uL
Hemoglobin	11.9	13.5-17.5 g/dL
Platelets	426	150K-450K/uL
Troponin	<3	<34ng/L
UA leukocytes	Moderate	negative
UA WBC	0-3	0-3 / hpf
UA RBC	3-6	0-2 / hpf
UA epithelial cells	many	NA
Venous pH	7.409	7.31-7.45
Venous pCO2	39.8	41-57 mm Hg
Fungitell quantitative value	<31	<60 pg/ml
HIV	Non-reactive	NA
FANA	1:40	<1:80
ANCA	Negative	NA
RF IgM	0.9	<4.9 IU/ml
Anti-MPO	<3.2	<20CU
Anti-PR3	<2.3	<20CU
CRP	3.4	<1.0 mg/dL

**Figure 1:**
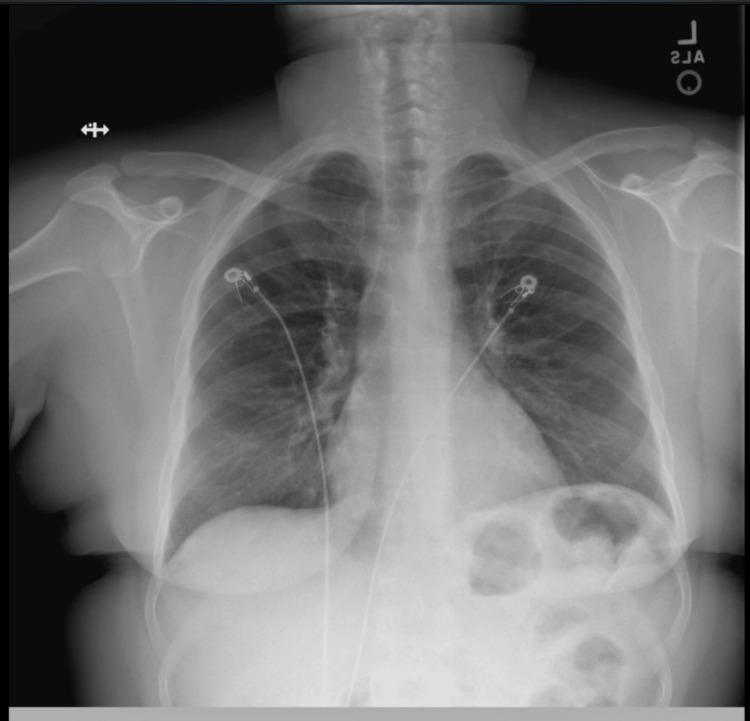
Chest radiograph demonstrating no acute cardiopulmonary findings on admission

**Figure 2:**
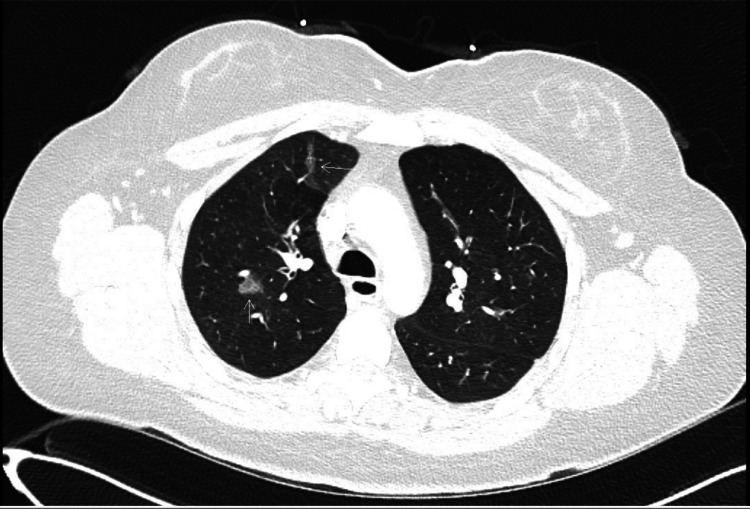
CT pulmonary angiography demonstrating multifocal bilateral ground-glass opacities with upper lobe predominance. Arrows indicate areas of ground-glass opacity CT: computed tomography

Subsequent testing revealed a normal *Aspergillus fumigatus* IgE and IgG, along with an elevated erythrocyte sedimentation rate (ESR) of 84 mm/hr and a markedly elevated total IgE of 4,701 kU/L (Table 2).

**Table 2:** Notable labs after admission including reference ranges IgE: immunoglobulin E, IgG: immunoglobulin G, ESR: erythrocyte sedimentation rate, WBC: white blood cell, NA: not applicable, BAL: bronchoalveolar lavage

Lab	Lab value	Reference range
*Aspergillus fumigatus* IgE	0.16	<0.70 kilounits/L
*Aspergillus fumigatus* IgG	18.6	<102 mg/L
ESR	84	0-20 mm/Hr
Total IgE	4701	0-127 kilounits/L
BAL WBC count	334	NA (mcL)
BAL percent neutrophils	35	NA (%)
BAL percent lymphocytes	4	NA (%)
BAL percent eosinophils	23	NA (%)

Bronchoscopy with BAL was then performed. Endoscopic findings included erythema of the superior segment of the right lower lobe with thick secretions. BAL analysis demonstrated 334 WBCs/µL, with a differential of 35% neutrophils, 4% lymphocytes, and 23% eosinophils. Respiratory culture, fungal culture, and fungal smear showed no growth. A pneumonia panel was negative. BAL testing for *Histoplasma*, *Blastomyces*, *Coccidioides*, and *Aspergillus* antigens was also negative. Cytology was negative for malignant cells but demonstrated a markedly elevated lipid-laden macrophage index (>150) (Figure 3).

**Figure 3:**
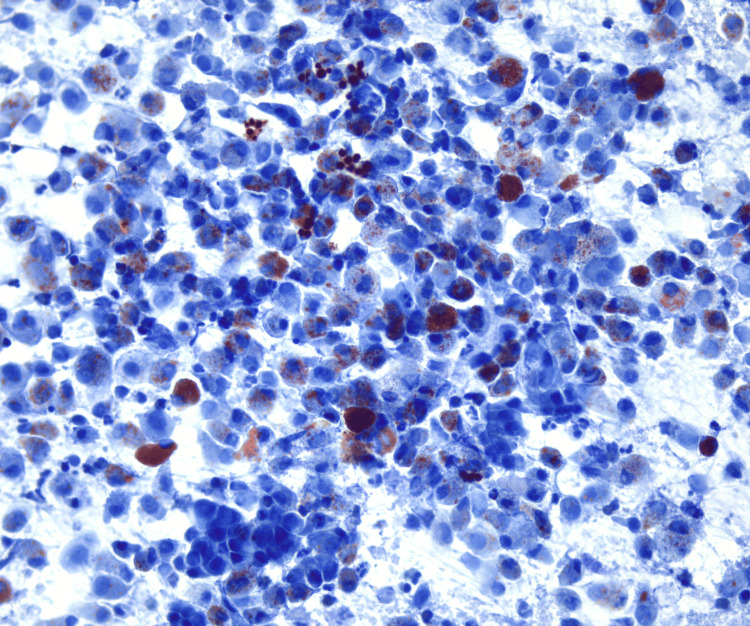
Oil Red O special stain at 40× magnification demonstrating numerous lipid-laden macrophages with >75% cytoplasmic opacification by lipid droplets staining red

She was started on a prednisone taper beginning at 60 mg daily. She was also initiated on trimethoprim-sulfamethoxazole (Bactrim) prophylaxis three times weekly. The patient was discharged on hospital day 4 with recommendations for outpatient pulmonology follow-up. Although the AEC remained elevated above normal at discharge, it had decreased compared with prior outpatient values obtained several months earlier (Figure 4).

**Figure 4:**
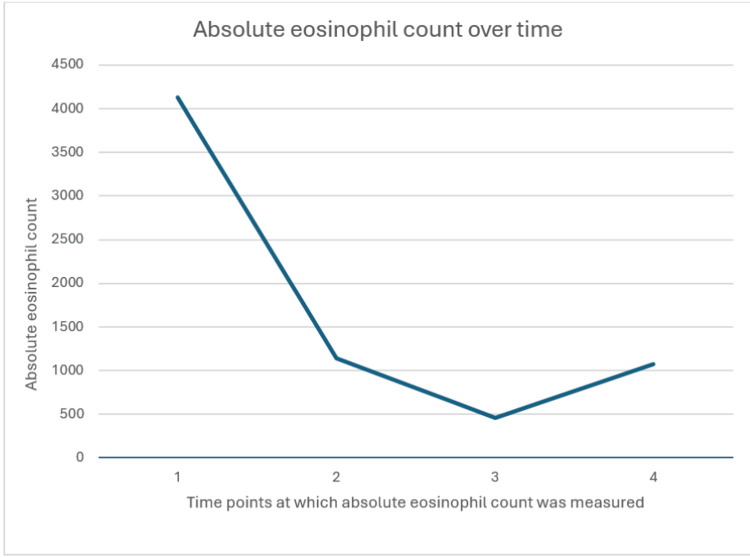
Line graph demonstrating AEC over time 1 = around two months prior to admission, 2 = at admission, 3 = two days after admission, 4 = at discharge AEC: absolute eosinophil count

A follow-up contrast-enhanced chest CT scan obtained 56 days after the initial CT pulmonary angiography protocol demonstrated complete resolution of the previously noted ground-glass opacities (Figure 5).

**Figure 5:**
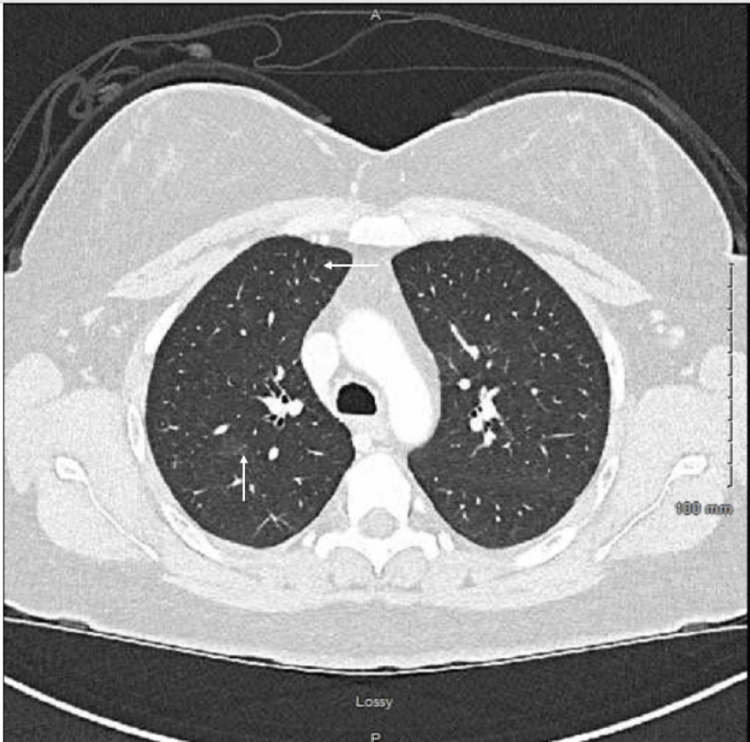
CT chest with IV contrast demonstrating resolution of the previously observed ground-glass opacities following completion of the patient’s steroid taper. Arrows indicate areas where ground-glass opacities were previously identified CT: computed tomography, IV: intravenous

## Discussion

Lipoid pneumonia is a rare form of lung disease resulting from aspiration or inhalation of exogenous or endogenous lipid-containing substances. It occurs when fat-containing material accumulates within the distal airways and alveoli, triggering an inflammatory response that impairs gas exchange [1,2]. Lipids are phagocytosed by alveolar macrophages, which are unable to metabolize them effectively. Following macrophage death, lipid material is repeatedly released into the alveolar spaces, perpetuating inflammation and, in some cases, leading to a granulomatous reaction.

The clinical presentation of exogenous lipoid pneumonia is often nonspecific and highly variable, ranging from asymptomatic disease to severe respiratory failure, including acute respiratory distress syndrome [1,10]. Common symptoms such as cough and dyspnea overlap with more prevalent pulmonary conditions, contributing to frequent misdiagnosis or delayed recognition [3,7]. The differential diagnosis includes pulmonary fibrosis, pulmonary alveolar proteinosis, bacterial pneumonia, viral pneumonia, and fungal pneumonia [2,3,5]. Consistent with previously reported cases, this patient underwent multiple healthcare visits and received empiric therapy prior to diagnosis, highlighting a well-described diagnostic challenge [3,11]. Radiologic findings are heterogeneous and may include consolidations, ground-glass opacities, or a “crazy paving” pattern, often mimicking infectious pneumonia, interstitial lung disease, or malignancy [1,12]. The multifocal ground-glass opacities observed in this case are consistent with prior reports of ongoing lipid exposure [2]. However, imaging findings alone are insufficient for diagnosis due to significant overlap with other disease processes.

Definitive diagnosis relies on histopathologic or cytologic identification of lipid-laden macrophages in respiratory secretions obtained via BAL or lung biopsy. Lipid-specific stains, such as Oil Red O or Sudan stain, may be used to confirm the presence of intracellular fat [6]. In this patient, BAL demonstrated a markedly elevated lipid-laden macrophage index, supporting the diagnosis. However, as previously described, this index has variable sensitivity and specificity and should be interpreted within the broader clinical context [13].

This case is notable for exposure to a commercially available bug spray containing plant-based oils. While exogenous lipoid pneumonia is most commonly associated with petroleum-based substances or aspiration of oil-based medications, there is growing recognition that aerosolized lipid products and plant-derived oils may also be causative agents [2,4]. Similar cases involving unconventional exposures, including herbal remedies, vaping products, and food oils, have been described, emphasizing the need for heightened clinical suspicion [4].

Management of exogenous lipoid pneumonia primarily involves cessation of exposure to the offending agent, which remains the cornerstone of therapy [3,4]. Corticosteroids have been used in selected cases to reduce inflammation. They may accelerate clinical and radiographic improvement by decreasing interstitial inflammation and improving lung aeration, allowing endogenous clearance mechanisms to recover [3,11,14-16]. In more severe or refractory cases, interventions such as bronchoscopic segmental lavage, whole-lung lavage, or surgical resection have been reported [8,17]. The patient in this case responded well to corticosteroid therapy and avoidance of further exposure, consistent with outcomes described in the literature [3,14].

From a clinical perspective, earlier recognition of a potential exposure-related etiology may have altered this patient’s diagnostic trajectory. A more detailed exposure history during initial presentations could have prompted earlier consideration of lipoid pneumonia, potentially reducing the need for repeated healthcare visits and unnecessary antibiotic use. Earlier bronchoscopy with BAL may also have expedited diagnosis. Delayed diagnosis in similar cases is well documented and often reflects the nonspecific nature of symptoms and imaging findings [12].

This case report has several limitations. As a single-patient observation, the findings are inherently limited in generalizability. Although the patient had documented exposure to a bug spray containing plant-based oils, the specific contribution of individual components to disease development cannot be definitively determined. Additionally, while BAL findings demonstrated lipid-laden macrophages consistent with lipoid pneumonia, this feature is not entirely specific and may be seen in other conditions associated with aspiration [9]. The markedly elevated IgE level may suggest alternative or coexisting disease processes, but does not exclude lipoid pneumonia. There is also the possibility of incomplete exposure history or recall bias, which may affect the identification of the inciting agent. Finally, corticosteroid therapy was initiated prior to definitive diagnosis, which may have altered the clinical presentation and radiographic evolution, thereby limiting assessment of the untreated natural disease course.

## Conclusions

Overall, this case reinforces the importance of maintaining a broad differential diagnosis in patients with persistent respiratory symptoms and atypical imaging findings. It also highlights the critical role of obtaining a detailed exposure history in patients with unexplained radiographic abnormalities. The case underscores the growing recognition of nontraditional lipid exposures, including plant-based aerosolized products, as potential etiologies of exogenous lipoid pneumonia.

In this patient, BAL cytology demonstrated lipid-laden macrophages, supporting lipoid pneumonia as the likely cause of her symptoms. She had a history of exposure to a bug spray containing cottonseed and clove oil. Recognition of lipoid pneumonia as a potential diagnosis in patients with a compatible exposure and clinical history can facilitate earlier identification and more targeted management.
